# Dose-dependent relationships in potential prescribing cascades: a cohort study using community pharmacy dispensing data

**DOI:** 10.1007/s11096-025-02083-y

**Published:** 2026-02-17

**Authors:** Ruveyda Gündogan-Yilmaz, Sadaf Wahedi, Johanna H. M. Driessen, Atiya Mohammad, Petra Denig, Fatma Karapinar-Carkit

**Affiliations:** 1 Department of Clinical Pharmacy, OLVG, Amsterdam, The Netherlands; 2 Department of Clinical Pharmacy and Toxicology, Maastricht University Medical Center+, Maastricht, The Netherlands; 3 Department of Clinical Pharmacy, CARIM, Cardiovascular Research Institute Maastricht, Maastricht University, Maastricht, The Netherlands; 4 Department of Clinical Pharmacy and Pharmacology, University Medical Centre Groningen, University of Groningen, Groningen, The Netherlands

**Keywords:** Drug-related adverse reactions, Drug prescriptions, Pharmacoepidemiology, Prescribing cascades, Polypharmacy

## Abstract

**Introduction:**

Prescribing cascades occur when new medications are initiated to treat adverse drug reactions (ADRs) caused by an initial medication (index). Although using lower dose of the index medication is often recommended as a general strategy to address adverse drug reactions that may trigger potential prescribing cascades, evidence supporting a dose-dependent relationship for many prescribing cascades is limited.

**Aim:**

The aim was to examine the dose-dependent relationship across a range of index medications related to various potential prescribing cascades, for which the dose-dependent relationship between the index medication and the ADR was not yet known.

**Method:**

A cohort study was conducted using prescription sequence symmetry analysis with data from over 600 Dutch community pharmacies (2015–2020). We assessed 18 potential prescribing cascades involving ACE inhibitors (ACEIs), statins, antidepressants, dihydropyridine calcium channel blockers (DCCBs), and other drug classes. Index medication doses were categorized based on the World Health Organization (WHO) Defined Daily Dose (DDD) into low (< 0.50 DDD), medium (0.50–1.50 DDD), and high (> 1.50 DDD). The adjusted sequence ratio (aSR) quantified the likelihood of marker drug initiation after vs. before the index drug, corrected for prescribing trends; aSR > 1 indicated a potential prescribing cascade.

**Results:**

Twelve of the 18 potential prescribing cascades showed a dose-dependent relationship. All angiotensin converting enzyme inhibitor (ACEI) related cascades demonstrated increasing aSRs with higher doses. ACEIs associated with cough showed increasing aSRs, from 0.86 to 2.09 at low dose to 1.29 to 2.78 at high dose across four cascades. Dose-dependent relationships were also found for statins, antidepressants, and DCCBs. No such relationship was observed for cascades involving proton pump inhibitors, diuretics, and non-steroidal anti-inflammatory drugs.

**Conclusion:**

Medication dose can play a significant role in potential prescribing cascades. Healthcare professionals should be aware of the potential contribution of dose to prescribing cascade development. The study design precludes causal inference, and confirmation is needed to support further clinical recommendations.

**Supplementary Information:**

The online version contains supplementary material available at 10.1007/s11096-025-02083-y.

## Impact statements

- Potential prescribing cascades may be dose-dependent, particularly for angiotensin-converting enzyme inhibitors, antidepressants and dihydropyridine calcium channel blockers.
- Before initiating new medication, attention should be paid to recent dose increases that can trigger an adverse drug reaction.
- Although a dose decrease may serve as an option to manage potential prescribing cascades, more research is needed to confirm the dose-response patterns.

## Introduction

A prescribing cascade occurs when an adverse drug reaction (ADR) from medication, referred to as the index medication, leads to the prescription of an additional medication, referred to as the marker medication, to treat that ADR [1]. Prescribing cascades contribute to polypharmacy, higher costs, and increased risk of new ADRs [2–4]. Recent studies have reported over 100 distinct prescribing cascades across different therapeutic areas, involving a wide variety of drug classes [5–7].

Strategies to manage prescribing cascades include stopping, substituting, or using lower doses of the initial medication [8, 9]. Discontinuation or substitution may not always be a viable option due to a lack of sufficiently effective alternatives or the risk of similar or new ADRs. Using a lower dose of the initial medication may prevent or reverse ADRs while preserving therapeutic effect. Despite general recommendations to lower doses to prevent or reverse ADRs and thereby prescribing cascades, evidence for dose-dependent relationships remains limited [10]. A systematic review showed that in 52 prescribing cascades authors advised using a lower dose. Yet, only 12 (23%) included a dose–response analysis to support this recommendation [10]. Moreover, cut-offs for defining “high dose” varied across studies [4].

Some single-cascade studies have reported higher rates of potential prescribing cascades at increased doses of medications [11–14]. A recent case study further illustrated this: dose escalation of an index drug led to ADRs, subsequent prescribing, and ultimately a hospital admission [15]. To provide guidance on how best to manage prescribing cascades, there is a need to evaluate dose-dependence across cascades, that have been identified as clinically relevant based on expert consensus regarding harm and frequency in practice [6].

### Aim

The aim was to examine the dose-dependent relationship across a range of index medications related to potential prescribing cascades for which the dose-dependent relationship between the medication and the ADR was not yet known.

## Method

### Setting

Medication data from January 1, 2015 to December 31, 2020 were collected from the Ncontrol database. Ncontrol includes data from > 600 community pharmacies located in urban and rural areas across the Netherlands (30% of all pharmacies) [6, 16]. It contains information on patient age and sex (male/female), and on dispensed medication, including medication name, Anatomical Therapeutic Chemical (ATC) code, strength and dosage scheme, and the start and end date of each dispensing. The end date is calculated as the dispensing date plus the recorded days’ supply, without adjusting for overlapping dispensings.

Patients in the Netherlands typically register at one pharmacy, where they collect all prescriptions. Dispensing data are commonly transferred when a patient switches between pharmacies to obtain an adequate medication history. Over-the-counter purchases in the pharmacy can be included but purchases elsewhere are not captured in the database.

### Study design

A cohort study was conducted to assess the dose-dependent relationship comparing low, medium, and high index drug doses. We applied prescription sequence symmetry analysis (PSSA) to calculate sequence ratios between index and marker medication [17]. The Strengthening the Reporting of Observational Studies in Epidemiology (STROBE) checklist for cohort studies was used to guide the reporting [18].

### Selection of problematic prescribing cascades

In a prior mixed-methods study, 66 cascades were deemed problematic by an expert panel (N = 16) including amongst others physicians and pharmacists. Two-third (N = 41) of these potential prescribing cascades showed a significant index–marker association in the Ncontrol database [6]. For these 41 potential prescribing cascades, we first examined whether there was evidence on dose dependence using a recent systematic review by Adrien et al. [10]. For cascades not clearly addressed in this review, we conducted additional searches in Micromedex® (IBM Watson Health, Greenwood Village, Colorado, USA), UpToDate® (2024 Wolters Kluwer N.V. and/or its subsidiaries), and PubMed [19, 20].

Prescribing cascades were included when no dose-dependence was reported or the evidence was inconsistent. We excluded those with fewer than 3,000 individuals using both index and marker drugs, as smaller numbers would not allow stable estimates across dose categories. This threshold was a pragmatic choice, and no formal power calculation was performed. In addition, potential prescribing cascades in which more than 10% of dispensing records involved stepwise dose reduction or dose increases were excluded. ATC codes for index and marker medications and details of excluded prescribing cascades are provided in Online Resource Tables S1–S3. Combination preparations were excluded for index medications, as it is unclear which component contributed to an ADR. For marker medications, combinations were retained, since their use reflects clinical management of the ADR irrespective of combination or single-agent therapy.

For validation, we included one positive control: dihydropyridine calcium channel blockers potentially inducing oedema followed by a diuretic, for which a dose–response relationship was confirmed in previous studies [2, 3].

### Patients included

Adult patients (≥ 18 years) were included who were dispensed the index and marker medication of interest and complied with four key PSSA parameters to identify a potential prescribing cascade [16]. First, a washout window of 12 months was applied to identify incident users. Second, the exposure window was set on 12 months ensuring that patients have the same follow-up period. Third, the maximum allowable gap between the expected end of an index medication and the dispensing of a marker medication (or vice versa) was set at four months. This so-called continued exposure interval (CEI) reduces the chance that marker initiation was unrelated to index medication use [21]. In the Dutch setting, dispensing records typically cover a maximum of three months; a four-month CEI therefore accounts for refill gaps and stockpiling, and aligns with previous recommendations based on pharmacoepidemiological cohort studies [16]. Finally, a blackout period of seven days was applied to both index-marker and marker-index sequences. This reflects the time after initiation during which events are not considered to be related, as an ADR leading to initiation of the marker medication within seven days is unlikely [21].

### Classification of dose categories

For each dispensing of the index medication, the daily dose was calculated by multiplying the strength with the number of units per day as specified in the dosage instructions. Individual dispensing records were excluded when the daily dose could not be calculated, for example, with a dosage scheme “use as needed”, or when the administration form was not oral. Patients remained in the analysis, unless all their dispensing records had missing dose information.

For each patient, the average daily dose of the index medication was calculated as the mean of all available dispensing records. For index-marker medication sequences, all dispensings available for the index medication from the first index medication dispensing up to the start of the marker medication were used within a maximum of 12 months. For marker-index medication sequences, all index medication dispensing records available within 12 months after the start of the marker medication were included. Next, three dose categories were set using the World Health Organization (WHO) defined daily dose (DDD) classification for the average daily dose of each index medication [22]. The dose categories were defined as follows: low > 0 and < 0.50 DDD, medium ≥ 0.50 and ≤ 1.50 DDD and high > 1.50 DDD. These cut-offs were chosen pragmatically, as has been done in previous research, to ensure consistency and comparability across cascades [23–25].

### Main outcome measure

The primary outcome is the adjusted sequence ratio (aSR) for each potential prescribing cascade.

### Analysis

PSSA was conducted to quantify the aSRs of the potential prescribing cascades across the dose categories. First, the crude sequence ratio (cSR) was calculated by dividing the total number of patients who initiated the index medication prior to the marker medication by the total number of patients who initiated the marker medication before the index medication [16]. Next, a null-effect sequence ratio (NSR) was calculated to account for prescribing trends [26]. Finally, the aSR was calculated by dividing the cSR by the NSR [27]. An aSR > 1 indicates the occurrence of a potential prescribing cascade [28, 29]. A dose-dependent relationship was considered to be confirmed when the aSR increased with higher dose categories and their 95% confidence intervals (CI) did not overlap [11, 14].

A sensitivity analysis was performed to investigate the robustness of our results by changing the cut-off values to ≤ 0.5 DDDs for low dose, > 0.5 and < 1.5 DDDs for medium dose, and ≥ 1.5 DDDs for high dose.

All analyses were carried out using IBM SPSS version 29 (IBM Corporation. Armonk. New York. U.S).

### Ethics approval

This study was approved by the local ethics committee of OLVG Hospital, Amsterdam, the Netherlands (Adviescommissie Wetenschappelijk Onderzoek-Medisch-Ethische Commissie, ACWO-MEC;registration number 20.132, April 12th, 2024).

## Results

In total, 18 potential prescribing cascades met our selection criteria (Online Resource Table S3), including the following drug classes as index medication: angiotensin converting enzyme inhibitors (ACEIs), statins, proton pump inhibitors (PPIs), diuretics, systemic non-steroidal anti-inflammatory drugs (NSAIDs), DCCBs, and antidepressants. The proportion of excluded dispensing records was < 2% for most cascades, 2–4.2% for two cascades, and 26.7% for NSAIDs due to frequent “as needed” use. The percentage of excluded patients was < 2% for most cascades, between 2 and 6% for six cascades and 27.7% for NSAIDs (Online Resource Table S4). Across all cascades, the mean age was lower in the high-dose-group (66.4 years) as compared to the low-dose-group (70.2 years). The proportion of women was generally lower in the high-dose groups compared to the low- and medium-dose groups (Tables 1 and 2).

**Table 1:** Characteristics for the positive control

Prescribing cascade	Total incident users (N =)	Women (n, %)*	Mean age (SD)	Index-Marker (N)	Marker-index (N)	Null-effect	cSR	aSR [95% CI]
*Dihydropyridine calcium channel blockers potentially causing oedema followed by high-ceiling diuretics (positive control)*								
Overall	10,113	5703 (56.4)	75.7 (12.4)	6667	3446	1.07	1.93	1.82 [1.77–1.86]
Low dose	168	108 (64.3)	76.6 (12.1)	104	64	1.06	1.63	1.53 [1.22–1.84]c
Medium dose	7229	4215 (58.3)	76.7 (11.8)	4634	2595	1.05	1.79	1.70 [1.65–1.74]b
High dose	2716	1380 (50.8)	73.0 (13.5)	1929	787	1.10	2.45	2.23 [2.14–2.31]b, c

cSR, crude sequence ratio; aSR, adjusted sequence ratio; n, number

^a^No overlap between low and medium dose

^b^No overlap between medium and high dose

^c^No overlap between low and high dose

^d^No overlap between all three dose-categories

*Percentages shown represent women; for less than 1% of the patients the sex was unknown, the remainder corresponds to men

**Table 2:** Potential prescribing cascades with angiotensin converting enzyme inhibitors (ACEIs), statins, and with other index medications

Prescribing cascade	Total incident users (N)	Women (N, %)^g^	Mean age (SD)	Index-marker (N)	Marker-index (N)	Null-effect	cSR	aSR [95% CI]
*ACEI potentially causing cough followed by inhaled adrenergics*								
Overall	14,927	7620 (51.0)	67.5 (13.5)	8308	6619	1.03	1.26	1.22 [1.19–1.25]
Low dose	715	381 (53.3)	72.9 (14.4)	328	387	0.99	0.85	0.86 [0.71–1.00]d
Medium dose	11,617	5962 (51.3)	67.2 (13.4)	6352	5265	1.02	1.21	1.18 [1.14–1.21]d
High dose	2595	1277 (49.2)	67.6 (13.2)	1628	967	1.07	1.68	1.57 [1.49–1.65]d
*ACEI potentially causing cough followed by antibacterials for systemic use*								
Overall	11,646	5641 (48.4)	68.1 (13.6)	8043	3603	1.11	2.23	2.00 [1.96–2.04]
Low dose	544	278 (51.1)	72.7 (14.6)	334	210	1.06	1.59	1.50 [1.32–1.67]d
Medium dose	9066	4446 (49.0)	68.0 (13.4)	6194	2872	1.11	2.16	1.94 [1.90–1.98]d
High dose	2036	917 (45.0)	67.7 (14.0)	1515	521	1.14	2.91	2.55 [2.45–2.65]d
*ACEI potentially causing cough followed by cough and cold preparations*								
Overall	19,173	10,342 (53.9)	68.4 (13.1)	14,215	4958	1.12	2.87	2.55 [2.52–2.58]
Low dose	942	532 (56.5)	73.4 (13.5)	655	287	1.09	2.28	2.09 [1.95–2.23]d
Medium dose	15,209	8319 (54.7)	68.3 (13.0)	11,259	3950	1.12	2.85	2.54 [2.50–2.58]d
High dose	3022	1491 (49.3)	67.3 (12.9)	2301	721	1.15	3.19	2.78 [2.70–2.86]d
*ACEI potentially causing cough followed by antihistamines for systemic use*								
Overall	11,475	6121 (53.3)	64.2 (14.1)	7294	4181	1.06	1.74	1.64 [1.61–1.68]
Low dose	552	303 (54.9)	68.5 (16.0)	336	216	1.07	1.56	1.45 [1.28–1.62]c
Medium dose	9132	4947 (54.2)	64.1 (14.0)	5695	3437	1.05	1.66	1.57 [1.53–1.62]b
High dose	1791	871 (48.6)	63.6 (14.0)	1263	528	1.10	2.39	2.18 [2.08–2.29]b, c
*ACEI potentially causing urinary tract infections followed by antibacterials for systemic use*								
Overall	32,673	16,842 (51.5)	69.4 (14.0)	21,997	10,676	1.09	2.06	1.89 [1.87–1.91]
Low dose	1683	907 (53.9)	74.5 (14.4)	1000	683	1.06	1.46	1.38 [1.29–1.48]d
Medium dose	25,329	13,174 (52.0)	69.1 (13.9)	16,847	8482	1.09	1.99	1.83 [1.80–1.86]d
High dose	5661	2761 (48.8)	68.9 (14.1)	4150	1511	1.12	2.75	2.44 [2.39–2.50]d
*ACEI potentially causing arthritis followed by NSAIDs/anti-rheumatics*^e^								
Overall	23,151	10,848 (46.9)	64.0 (12.8)	13,594	9557	1.06	1.42	1.34 [1.32–1.37]
Low dose	811	418 (51.5)	67.6 (14.3)	449	362	1.04	1.24	1.19 [1.05–1.33]c
Medium dose	18,306	8696 (47.5)	63.9 (12.7)	10,547	7759	1.05	1.36	1.29 [1.26–1.32]b
High dose	4034	1734 (43.0)	63.8 (12.8)	2598	1436	1.09	1.81	1.65 [1.59–1.72]b, c
*ACEI potentially causing depression followed by antidepressants*^f^								
Overall	9719	5293 (54.5)	65.9 (14.2)	5210	4509	1.02	1.16	1.13 [1.09–1.17]
Low dose	461	271 (58.8)	71.0 (15.5)	232	229	1.02	1.01	1.00 [0.81–1.18]c
Medium dose	7513	4128 (54.9)	65.7 (14.1)	3974	3539	1.02	1.12	1.10 [1.06–1.15]b
High dose	1745	894 (51.2)	65.2 (13.7)	1004	741	1.05	1.35	1.29 [1.20–1.39]b, c
*Statins potentially causing erectile dysfunction followed by drugs for erectile dysfunction*								
Overall	4933	20 (0.4)	65.3 (9.6)	3267	1666	1.08	1.96	1.81 [1.75–1.87]
Low dose	181	1 (0.6)	67.2 (9.5)	119	62	1.10	1.92	1.75 [1.44–2.06]
Medium dose	3857	15 (0.4)	65.4 (9.5)	2531	1326	1.08	1.91	1.76 [1.70–1.83]b
High dose	895	4 (0.4)	64.4 (9.8)	617	278	1.10	2.22	2.02 [1.88–2.17]b
*Statins potentially causing urinary incontinence followed by drugs for incontinence*								
Overall	11,808	2066 (17.5)	71.7 (10.2)	7072	4736	1.05	1.49	1.42 [1.38–1.46]
Low dose	487	101 (20.7)	71.9 (10.4)	272	215	1.06	1.27	1.20 [1.02–1.37]a
Medium dose	9382	1718 (18.3)	71.7 (10.3)	5628	3754	1.05	1.50	1.42 [1.38–1.46]a
High dose	1939	247 (12.7)	71.6 (9.8)	1172	767	1.06	1.53	1.45 [1.36–1.54]
*Statins potentially causing depression followed by antidepressants*^f^								
Overall	16,787	9211 (54.9)	65.2 (12.5)	9227	7561	1.03	1.22	1.18 [1.15–1.21]
Low dose	707	466 (65.9)	67.1 (12.7)	357	350	1.02	1.02	1.00 [0.85–1.15]c
Medium dose	13,477	7547 (56.0)	65.3 (12.5)	7405	6073	1.03	1.22	1.18 [1.15–1.22]
High dose	2603	1198 (46.0)	64.4 (12.6)	1465	1138	1.04	1.29	1.24 [1.16–1.32]c
*Statins potentially causing agitation followed by antipsychotics / benzodiazepines*								
Overall	25,748	14,102 (54.8)	67.0 (12.7)	15,124	10,624	1.05	1.42	1.35 [1.33–1.38]
Low dose	1196	763 (63.8)	70.1 (12.2)	709	487	1.06	1.46	1.37 [1.25–1.48]
Medium dose	20,485	11,490 (56.1)	67.1 (12.7)	12,024	8461	1.05	1.42	1.35 [1.32–1.38]
High dose	4067	1849 (45.5)	66.1 (12.6)	2391	1676	1.05	1.43	1.36 [1.30–1.42]
*Statins potentially causing confusion followed by antipsychotics*								
Overall	4524	2198 (48.6)	65.2 (15.0)	2586	1938	1.04	1.33	1.28 [1.22–1.34]
Low dose	170	93 (54.7)	67.9 (15.2)	100	70	1.04	1.43	1.38 [1.07–1.68]
Medium dose	3708	1838 (49.6)	65.2 (15.0)	2099	1609	1.04	1.30	1.25 [1.19–1.32]
High dose	646	267 (41.3)	64.8 (14.6)	387	259	1.05	1.49	1.42 [1.26–1.58]
*Statins potentially causing sleeplessness followed by hypnotics/sedatives*								
Overall	15,033	8228 (54.7)	67.8 (12.6)	9088	5945	1.06	1.53	1.45 [1.41–1.48]
Low dose	652	412 (63.2)	69.9 (12.6)	386	266	1.07	1.45	1.35 [1.20–1.51]
Medium dose	12,061	6834 (56.7)	68.0 (12.7)	7295	4766	1.06	1.53	1.45 [1.41–1.48]
High dose	2320	982 (42.3)	66.4 (12.2)	1407	913	1.05	1.54	1.46 [1.38–1.55]
*Antidepressants potentially causing urinary incontinence followed by drugs for incontinence*								
Overall	6217	2210 (35.5)	66.2 (16.2)	3248	2969	1.01	1.09	1.08 [1.03–1.13]
Low dose	2498	907 (36.3)	69.0 (15.4)	1098	1400	0.98	0.78	0.80 [0.72–0.88]d
Medium dose	3299	1141 (34.6)	65.1 (16.4)	1859	1440	1.02	1.29	1.26 [1.19–1.33]d
High dose	420	162 (38.6)	57.9 (15.5)	291	129	1.12	2.26	2.02 [1.81–2.23]d
*Dihydropyridine calcium channel blockers potentially causing depression followed by antidepressants*^f^								
Overall	8849	5356 (60.5)	67.8 (13.8)	4702	4147	1.02	1.13	1.12 [1.07–1.16]
Low dose	178	136 (76.4)	63.7 (15.0)	83	95	1.04	0.87	0.84 [0.55–1.13]c
Medium dose	6742	4153 (61.6)	68.2 (13.7)	3530	3212	1.01	1.10	1.09 [1.04–1.14]b
High dose	1929	1067 (55.3)	66.6 (13.9)	1089	840	1.04	1.30	1.25 [1.16–1.34]b, c
*Thiazide & loop diuretics potentially causing diuresis followed by urologicals*								
Overall	3201	1837 (57.4)	75.4 (12.1)	1741	1460	1.04	1.19	1.15 [1.08–1.22]
Low dose	296	157 (53.0)	76.6 (11.4)	178	118	1.07	1.51	1.41 [1.18–1.65]
Medium dose	2098	1262 (60.2)	74.2 (12.3)	1140	958	1.04	1.19	1.14 [1.06–1.23]
High dose	807	418 (51.8)	78.2 (11.2)	423	384	1.02	1.10	1.08 [0.94–1.21]
*Systemic NSAIDs potentially causing peripheral oedema followed by high-ceiling diuretics*								
Overall	8170	5259 (64.4)	73.3 (14.0)	4407	3763	1.01	1.17	1.16 [1.12–1.21]
Low dose	233	174 (74.7)	70.7 (15.4)	143	90	1.08	1.59	1.47 [1.20–1.73]
Medium dose	5587	3627 (64.9)	74.3 (14.0)	2978	2609	1.00	1.14	1.14 [1.09–1.20]
High dose	2350	1458 (62.0)	71.2 (13.5)	1286	1064	1.01	1.21	1.19 [1.11–1.27]
*Proton pump inhibitors potentially causing infection followed by intestinal anti-infectives*								
Overall	7909	5233 (66.2)	62.6 (16.3)	5810	2099	1.10	2.77	2.51 [2.46–2.56]
Low dose	8	7 (87.5)	68.9 (18.4)	6	2	1.27	3.00	2.36 [0.76–3.96]
Medium dose	5486	3634 (66.2)	63.6 (16.4)	4051	1435	1.10	2.82	2.56 [2.50–2.62]
High dose	2415	1592 (65.9)	60.4 (16.0)	1753	662	1.10	2.65	2.40 [2.31–2.49]

cSR, crude sequence ratio; aSR, adjusted sequence ratio; SD, standard deviation; ACEI, angiotensin-converting enzyme inhibitor; NSAID, non-steroidal anti-inflammatory drug

^a^No overlap between low and medium dose

^b^No overlap between medium and high dose

^c^No overlap between low and high dose

^d^No overlap between all three dose-categories

^e^Antidepressants include N06AA, N06AB, N06AX

^f^NSAIDs/anti-rheumatics include M01A

^g^Percentages shown represent women; for less than 1% of the patients the sex was unknown, the remainder corresponds to men

The positive control showed a step-wise increase in aSR between the low and high dose groups, with no overlapping CIs (Table 1).

**Fig. 1:**
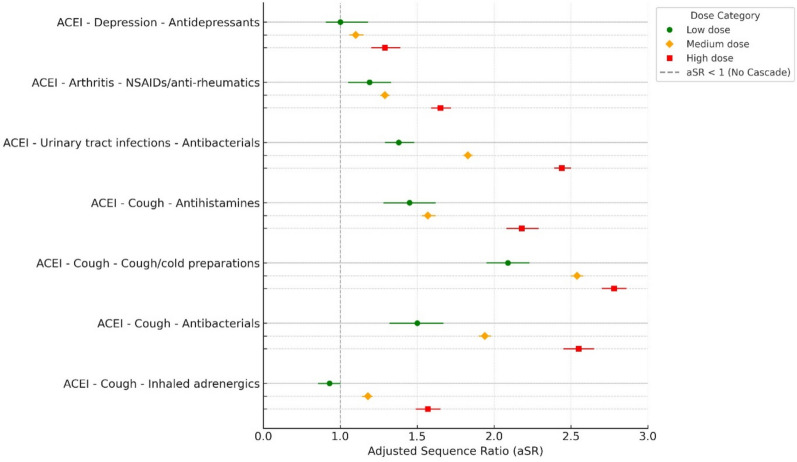
Adjusted sequence ratios (aSRs) with 95% confidence intervals for seven angiotensin-converting enzyme inhibitor (ACEI)-related potential prescribing cascades by dose category. Each cascade is shown with three colored markers: green circle for low dose, orange diamond for medium dose, and red square for high dose. The dashed vertical line at aSR = 1 represents the null effect (no cascade). ACEI = angiotensin-converting enzyme inhibitor, NSAID = non-steroidal anti-inflammatory drug. Marker drug NSAIDs/anti-rheumatics includes M01A only

Seven prescribing cascades had ACEIs as index medication, all with an overall aSR > 1. A dose-dependent relationship was observed across all ACEI cascades (Fig. 1, Table 2). Four showed a step-wise increase in aSRs from the low to the medium and from the medium to the high-dose-group (i.e., ACEI potentially causing cough followed by inhaled adrenergics or by antibacterials for systemic use or by cold preparations and ACE potentially causing urinary tract infections followed by antibacterials for urinary tract infections). For the other three cascades (i.e., ACEI potentially causing arthritis, depression or cough followed by NSAIDs, antidepressants or antihistamines for systemic use, respectively) the high-dose-group showed a step-wise increase in aSR without overlap in CI in comparison to the low and medium-dose-groups. In two cases, the aSR became non-significant for the low-dose-group.

**Fig. 2:**
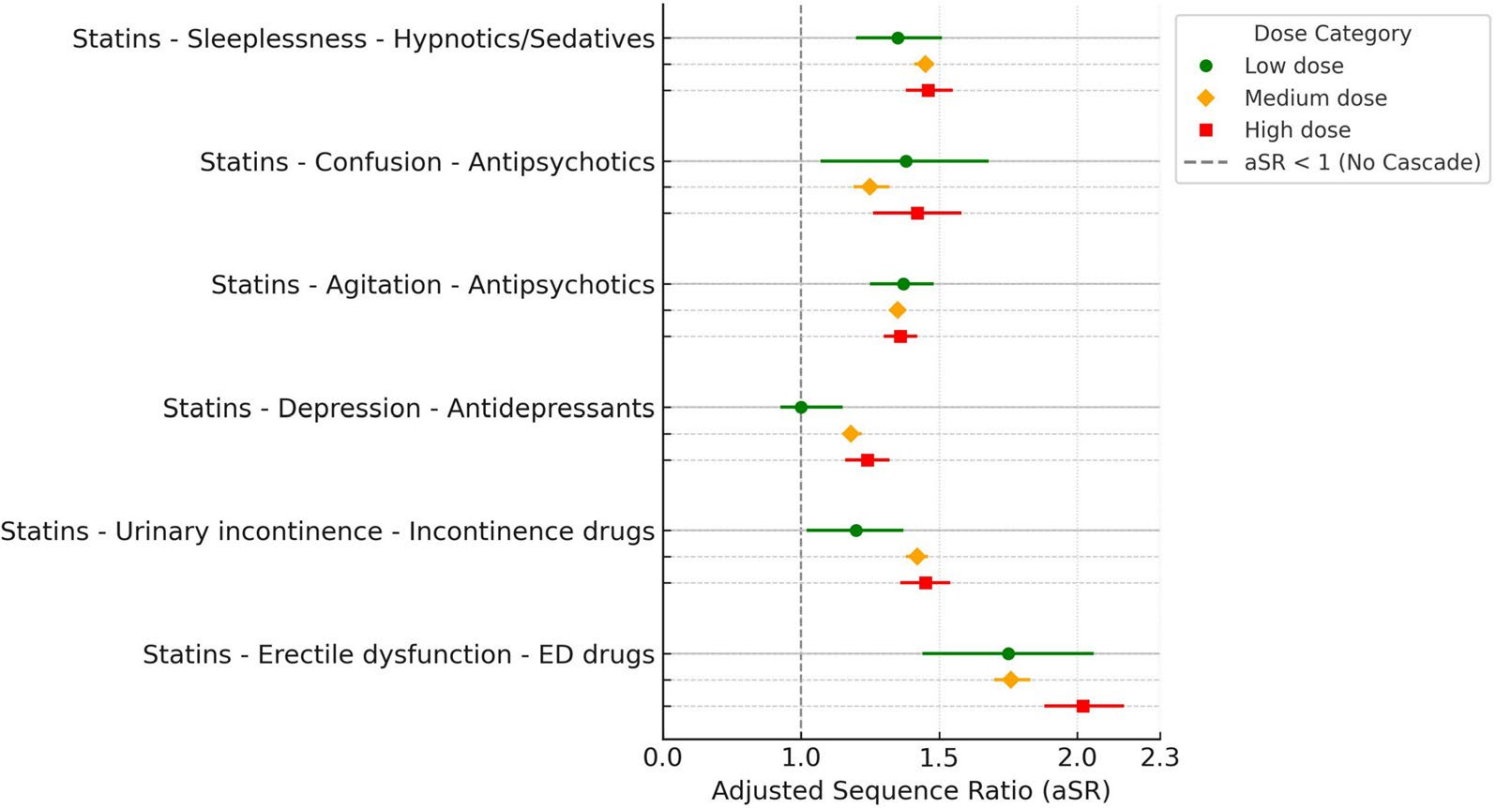
Adjusted sequence ratios (aSRs) with 95% confidence intervals for six statin-related prescribing cascades by dose category. Each cascade is represented with three colored markers: green for low dose, orange for medium dose, and red for high dose. Two statin-related cascades related to antipsychotics were analysed: agitation (marker drugs: antipsychotics [N05A excl. N05AN] and benzodiazepines [N05BA, N05CD]) and confusion (marker drugs: antipsychotics only). The dashed vertical line at aSR = 1 represents the null effect (no cascade). ED: erectile dysfunction

Statins were included for six prescribing cascades, three of which showed a dose-dependent relationship. One cascade, statins potentially causing erectile dysfunction (ED) followed by medication for ED, showed a step-wise increase in aSR between the medium and high-dose-groups without overlap in CI (Fig. 2, Table 2). Another cascade, statins potentially causing urinary incontinence followed by medications for urinary frequency and incontinence, showed a step-wise increase in aSR for the medium dose-group in comparison with the low-dose-group. A third cascade, statins potentially causing depression followed by antidepressants, showed a step-wise increase in aSR for the high-dose-group without overlap in CI in comparison to the low-dose-group.

**Fig. 3:**
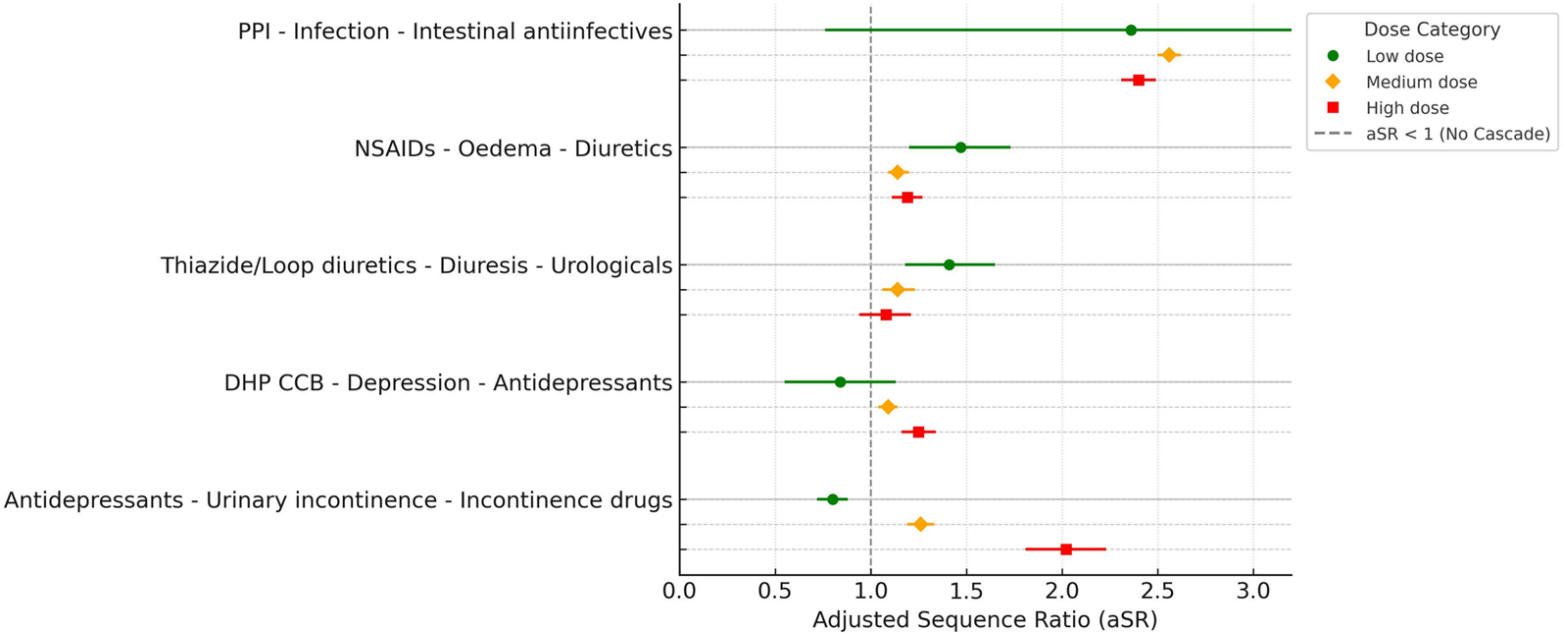
Adjusted sequence ratios (aSRs) with 95% confidence intervals for prescribing cascades involving antidepressants, dihydropyridine calcium channel blockers (DHP CCBs), diuretics, non-steroidal anti-inflammatory drugs (NSAIDs), and proton pump inhibitors (PPIs) by dose category. Each cascade is represented with three colored markers: green for low dose, orange for medium dose, and red for high dose. The dashed vertical line at aSR = 1 represents the null effect (no cascade). PPI: proton pump inhibitors. NSAIDS: non-steroidal anti-inflammatory drugs. DHP CCB: dihydropyridine calcium channel blockers

Antidepressants were included for one prescribing cascade, potentially causing increased urinary frequency and incontinence. This cascade showed a step-wise increase in aSR for the low, medium and high-dose-groups without overlap in CI (Fig. 3, Table 2). The prescribing cascade for DCCBs, potentially causing depression followed by antidepressants, showed a step-wise increase in aSR for the high-dose-group as compared to the low and medium-dose-groups. In both cases, the aSR became non-significant for the low-dose-group (Fig. 3, Table 2).

No dose dependent relationship was found for three remaining potential prescribing cascades (Fig. 3, Table 2).

The results of our sensitivity analysis did not change the point estimates substantially (Online Resource Table S5). The prescribing cascade involving ACEI potentially causing cough followed by antihistamines for systemic use now showed a step-wise increase in aSR for the low, medium and high-dose-groups without overlap in CI. Two potential prescribing cascades involving statins, with marker medications for urinary incontinence or antidepressants, now showed overlap between all three dose groups.

## Discussion

### Statement of key findings

In this large cohort study, we evaluated 18 potential prescribing cascades, of which 12 demonstrated a dose-dependent relationship and 10 still showed this relationship in the sensitivity analyses. All ACEI-related cascades consistently showed higher aSRs at higher doses, while dose-dependence was also observed for several statin-, antidepressant- and DCCB-related cascades. In contrast, no clear dose-dependent associations were found for potential prescribing cascades involving PPIs, diuretics, or NSAIDs.

### Comparison with previous literature

ACEI-induced cough has not been reported as a dose-related ADR in general reference materials [30, 31]; however, one trial has suggested a possible dose-dependent relationship for ramipril [32]. Our study showed a notable increase in aSRs corresponding to higher doses of ACEI for all cough-related marker medications tested. Although switching to an ARB is commonly advised when patients develop ACEI-induced cough, our study indicates that using a lower dose may be an alternative option when a switch is not preferred. Furthermore, our study indicates that other ACEI-related prescribing cascades are dose-dependent. It has been hypothesised that ACEI-induced urinary tract infections (UTIs) may be dose-dependent [33]. This could be due to the hemodynamic effects of ACEI on renal perfusion and filtration, which are more pronounced at higher dosages [34]. Since bacterial clearance from the urinary tract is dependent on urine output [35], it is plausible that ACEI use could be associated with an increased risk of UTIs, particularly at higher doses. For depression and arthritis, the underlying mechanism is unclear and there is conflicting evidence on whether ACEIs can induce these conditions [36, 37]. One study concluded that specific classes of antihypertensives, such as beta-blockers and DCCBs, are associated with increased risk of depression [38]. For antidepressants potentially causing urinary incontinence, there has been a case report supporting a dose-dependent relationship for sertraline-induced urinary incontinence [39]. Antidepressants may contribute to urinary incontinence by causing urinary retention, which may lead to overflow incontinence, and by inhibiting cholinergic, adrenergic, and histaminergic receptors [40].

### Strengths and limitations

We used data from over 600 Dutch pharmacies, representing one-third of all national pharmacies [16]. We calculated average daily dose based on the dispensing records per patient using the WHO DDD classification system, which has been developed to account for differences in potency within medication classes [22]. We included a positive control and conducted sensitivity analyses, since we used arbitrary cut-off values which may not fully correspond with the doses of available products. The sensitivity analyses showed that two of the potential prescribing cascades involving statins lost a dose-dependent relationship.

This study has several limitations. First, for the NSAID cascade, 26.7% of dispensing records lacked dose information due to the frequent use of 'as needed' regimens. This substantial proportion of missing data may have obscured potential dose-dependent associations. For most cascades (15/18), the percentage of missing was less than 2%.

Second, dispensing data may not fully represent medication exposure. Calculating the average dose per dispensing can overestimate actual exposure when a patient is non-adherent to the medication, which would lower the likelihood of detecting a dose-dependent relationship. In addition, we combined different medications in classes which masks potential differences at substance level. Also, by combining, for example, loop diuretics with thiazide diuretics the dose-dependent relationship with urologicals may have been diminished.

Another limitation follows from using the PSSA methodology, which does not adjust for all possible confounding. The absence of comorbidity data restricted our ability to adjust for confounding by indication. High doses of index medication may be associated with comorbidity leading to prescribing of the marker medication, for example in case of depression [41]. High doses can also be associated with more co-medication potentially causing the same ADR, for example in case of erectile dysfunction [42]. As a result, dose-dependent associations may be overestimated. Furthermore, we observed that lower doses were dispensed more in high age groups and women, who may be more susceptible to ADRs [43]. Such prescribing practices would diminish our ability to detect dose-dependent relationships. We did not perform stratified analyses by age or sex, as the focus of this study was on dose-dependence. Nonetheless, age and sex can be potential confounders, where differences in these characteristics between dose groups could partly explain observed associations. Finally, particularly low dose NSAIDs, cough medication and antihistamines over-the-counter purchases may have been missed, potentially impacting three prescribing cascades.

### Clinical implications and future research

Our findings indicate that dose can play a role in the occurrence of potential prescribing cascades. Although potential prescribing cascades at higher doses may be confounded with more complex underlying diseases, patient characteristics or co-medication, clinicians should be aware that high doses may contribute to symptoms of, for example, depression, ED or urinary incontinence. Such symptoms can arise with increasing dose but also with ageing. Vigilance is needed particularly when doses are increased over time, since previous research showed that patients and healthcare professionals did not consider the possibility of an ADR when patients have used medication for a long time [8]. If these findings are confirmed in other studies, they could also inform clinical decision support tools by flagging high-dose prescriptions that increase cascade risk, and could be incorporated into deprescribing protocols or guideline recommendations as triggers to consider dose adjustment before adding new therapies.

Future research should validate and extend our results among other populations and settings. More research is needed to examine the optimal method for studying dose-dependence of prescribing cascades, focusing on adjustment for confounding. Since results will depend on prescribing habits and the healthcare professionals’ recognition and management of ADRs, similar studies should be conducted in different countries and healthcare settings. For some potential prescribing cascades, larger datasets on low, medium, and high doses are needed to assess dose-dependent relationships. Finally, research is needed to examine the effectiveness of using a lower dose in alleviating or reversing ADRs and thereby potential prescribing cascades.

## Conclusion

This study indicates that several potential prescribing cascades initiated by ACEIs, antidepressants and DCCBs can be dose-dependent. In these cases, using a lower dose might be one of the strategies to prevent or reverse a potential prescribing cascade. However, the current study design limits causal interpretation, underscoring the need for further studies to confirm the findings of this study.

## Supplementary Information

Below is the link to the electronic supplementary material.

Supplementary file1 (DOCX 52 kb)

## Data Availability

The data that support the findings of this study were obtained from the NControl database, which contains anonymized dispensing data from Dutch community pharmacies. Due to contractual agreements and data protection regulations, these data are not publicly available. Access may be granted on reasonable request to NControl, subject to applicable ethical and legal approvals.
