# Supplementary material for: Dose-dependent relationships in potential prescribing cascades: a cohort study using community pharmacy dispensing data
Source: Int J Clin Pharm. 2026 Feb 17;48(3):874–85. doi: 10.1007/s11096-025-02083-y (PMC13176057; doi:10.1007/s11096-025-02083-y)
Supplement: Supplementary file 1 — Supplementary file1 (DOCX 52 kb) [file 11096_2025_2083_MOESM1_ESM.docx]

**Online Resource Table S1. Overview of ATC codes used to identify systemic index medications and the used dose cut-off values.**

| **ATC-Codeᵃ** | **Drug** | **DDDᵇ** | **Low dose** | **Medium dose** | **High dose** |
| --- | --- | --- | --- | --- | --- |
| **Proton Pump inhibitors (PPIs)** | |  |  |  |  |
| A02BC01 | omeprazole | 20 mg | < 10 mg | ≥ 10 - ≤ 30 mg | > 30 mg |
| A02BC02 | pantoprazole | 40 mg | < 20 mg | ≥ 20 - ≤ 60 mg | > 60 mg |
| A02BC03 | lansoprazole | 30 mg | < 15 mg | ≥ 15 - ≤ 45 mg | > 45 mg |
| A02BC04 | rabeprazole | 20 mg | < 10 mg | ≥ 10 - ≤ 30 mg | > 30 mg |
| A02BC05 | esomeprazole | 30 mg | < 15 mg | ≥ 15 - ≤ 45 mg | > 45 mg |
| **Diuretics** |  |  |  |  |  |
| C03AA03 | hydrochlorothiazide | 25 mg | < 12.5 mg | ≥ 12.5 - ≤ 37.5 mg | > 37.5 mg |
| C03BA04 | chlortalidone | 25 mg | < 12.5 mg | ≥ 12.5 - ≤ 37.5 mg | > 37.5 mg |
| C03BA11 | indapamide | 2.5 mg | < 1.25 mg | ≥ 1.25 - ≤ 3.75 mg | > 3.75 mg |
| C03CA01 | furosemide | 40 mg | < 20 mg | ≥ 20 - ≤ 30 mg | > 30 mg |
| C03CA02 | bumetanide | 1 mg | < 0.5 mg | ≥ 0.5 - ≤ 1.5 mg | > 1.5 mg |
| C03DA01 | spironolactone | 75 mg | < 37.5 mg | ≥ 37.5 - ≤ 112.5 mg | > 112.5 mg |
| C03DA04 | eplerenone | 50 mg | < 25 mg | ≥ 25 - ≤ 75 mg | > 75 mg |
| C03DB01 | amiloride | 10 mg | < 5 mg | ≥ 5 - ≤ 15 mg | > 15 mg |
| C03DB02 | triamterene | 100 mg | < 50 mg | ≥ 50 - ≤ 150 mg | > 150 mg |
| **Angiotensin Converting Enzyme Inhibitors (ACEIs)** | |  |  |  |  |
| C09AA01 | captopril | 50 mg | < 25 mg | ≥ 25 - ≤ 75 mg | > 75 mg |
| C09AA02 | enalapril | 10 mg | < 5 mg | ≥ 5 - ≤ 15 mg | > 15 mg |
| C09AA03 | lisinopril | 10 mg | < 5 mg | ≥ 5 - ≤ 15 mg | > 15 mg |
| C09AA04 | perindopril | 4 mg | < 2 mg | ≥ 2 - ≤ 6 mg | > 6 mg |
| C09AA05 | ramipril | 2.5 mg | < 1.25 mg | ≥ 1.25 - ≤ 3.75 mg | > 3.75 mg |
| C09AA06 | quinapril | 15 mg | < 7.5 mg | ≥ 7.5 - ≤ 22.5 mg | > 22.5 mg |
| C09AA07 | benazepril | 7.5 mg | < 3.75 mg | ≥ 3.75 - ≤ 11.25 mg | > 11.25 mg |
| C09AA09 | fosinopril | 15 mg | < 7.5 mg | ≥ 7.5 - ≤ 22.5 mg | > 22.5 mg |
| C09AA15 | zofenopril | 30 mg | < 15 mg | ≥ 15 - ≤ 45 mg | > 45 mg |
| **Nonsteroidal anti-inflammatory drugs (NSAIDs)** | | |  |  |  |
| M01AA01 | phenylbutazone | 300 mg | < 150 mg | ≥ 150- ≤ 450 mg | > 450 mg |
| M01AB01 | indomethacin | 100 mg | < 50 mg | ≥ 50 - ≤ 150 mg | > 150 mg |
| M01AB05 | diclofenac | 100 mg | < 50 mg | ≥ 50 - ≤ 150 mg | > 150 mg |
| M01AB16 | aceclofenac | 200 mg | < 100 mg | ≥ 100 - ≤ 300 mg | > 300 mg |
| M01AC01 | piroxicam | 20 mg | < 10 mg | ≥ 10 - ≤ 30 mg | > 30 mg |
| M01AC06 | meloxicam | 15 mg | < 7.5 mg | ≥ 7.5 - ≤ 22.5 mg | > 22.5 mg |
| M01AE01 | ibuprofen | 1200 mg | < 600 mg | ≥ 600 - ≤ 1800 mg | > 1800 mg |
| M01AE02 | naproxen | 500 mg | < 250 mg | ≥ 250 - ≤ 750 mg | > 750 mg |
| M01AE11 | tiaprofenic acid | 600 mg | < 300 mg | ≥ 300 - ≤ 900 mg | > 900 mg |
| M01AH01 | celecoxib | 200 mg | < 100 mg | ≥ 100 - ≤ 300 mg | > 300 mg |
| M01AH05 | etoricoxib | 60 mg | < 30 mg | ≥ 30 - ≤ 90 mg | > 90 mg |
| M01AX01 | nabumetone | 1000 mg | < 500 mg | ≥ 500 - ≤ 1500 mg | > 1500 mg |
| M01AX05 | glucosamine | 1500 mg | < 750 mg | ≥ 750 - ≤ 2250 mg | > 2250 mg |
| **Dihydropyridines** |  |  |  |  |  |
| C08CA01 | amlodipine | 5 mg | < 2.5 mg | ≥ 2.5 - ≤ 7.5 mg | > 7.5 mg |
| C08CA02 | felodipine | 5 mg | < 2.5 mg | ≥ 2.5 - ≤ 7.5 mg | > 7.5 mg |
| C08CA03 | isradipine | 5 mg | < 2.5 mg | ≥ 2.5 - ≤ 7.5 mg | > 7.5 mg |
| C08CA05 | nifedipine | 30 mg | < 15 mg | ≥ 15 - ≤ 45 mg | > 45 mg |
| C08CA06 | nimodipine | 300 mg | < 150 mg | ≥ 150 - ≤ 450 mg | > 450 mg |
| C08CA08 | nitrendipine | 20 mg | < 10 mg | ≥ 10 - ≤ 30 mg | > 30 mg |
| C08CA09 | lacidipine | 4 mg | < 2 mg | ≥ 2 - ≤ 6 mg | > 6 mg |
| C08CA12 | barnidipine | 10 mg | < 5 mg | ≥ 5 - ≤ 15 mg | > 15 mg |
| C08CA13 | lercanidipine | 10 mg | < 5 mg | ≥ 5 - ≤ 15 mg | > 15 mg |
| **Antidepressants** |  |  |  |  |  |
| N06AA02 | imipramine | 100 mg | < 50 mg | ≥ 50 - ≤ 150 mg | > 150 mg |
| N06AA04 | clomipramine | 100 mg | < 50 mg | ≥50 - ≤ 150 mg | > 150 mg |
| N06AA09 | amitriptyline | 75 mg | < 37.5 mg | ≥ 37.5 -≤ 112.5 mg | > 112.5 mg |
| N06AA10 | nortriptyline | 75 mg | < 37.5 mg | ≥ 37.5 - ≤ 112.5 mg | > 112.5 mg |
| N06AA12 | doxepin | 100 mg | < 50 mg | ≥ 50 - ≤ 150 mg | > 150 mg |
| N06AA16 | dosulepin | 150 mg | < 75 mg | ≥ 75 - ≤ 225 mg | > 225 mg |
| N06AA21 | maprotiline | 100 mg | < 50 mg | ≥ 50 - ≤ 150 mg | > 150 mg |
| N06AB03 | fluoxetine | 20 mg | < 10 mg | ≥ 10 - ≤ 30 mg | > 30 mg |
| N06AB04 | citalopram | 20 mg | < 10 mg | ≥ 10 - ≤ 30 mg | > 30 mg |
| N06AB05 | paroxetine | 20 mg | < 10 mg | ≥ 10 - ≤ 30 mg | > 30 mg |
| N06AB06 | sertraline | 50 mg | < 25 mg | ≥ 25 - ≤ 75 mg | > 75 mg |
| N06AB08 | fluvoxamine | 100 mg | < 50 mg | ≥ 50 - ≤ 150 mg | > 150 mg |
| N06AB10 | escitalopram | 10 mg | < 5 mg | ≥ 5 - ≤ 15 mg | > 15 mg |
| N06AF03 | phenelzine | 60 mg | < 30 mg | ≥ 30 - ≤ 90 mg | > 90 mg |
| N06AF04 | tranylcypromine | 10 mg | < 5 mg | ≥ 5 - ≤ 15 mg | > 15 mg |
| N06AX03 | mianserin | 60 mg | < 30 mg | ≥ 30 - ≤ 90 mg | > 90 mg |
| N06AX05 | trazodone | 300 mg | < 150 mg | ≥ 150 - ≤ 450 mg | > 450 mg |
| N06AX11 | mirtazapine | 30 mg | < 15 mg | ≥ 15 - ≤ 45 mg | > 45 mg |
| N06AX12 | bupropion | 300 mg | < 150 mg | ≥ 150 - ≤ 450 mg | > 450 mg |
| N06AX16 | venlafaxine | 100 mg | < 50 mg | ≥ 50 - ≤ 150 mg | > 150 mg |
| N06AX21 | duloxetine | 60 mg | < 30 mg | ≥ 30 - ≤ 90 mg | > 90 mg |
| N06AX22 | agomelatine | 25 mg | < 12.5 mg | ≥ 12.5 - ≤ 37.5 mg | > 37.5 mg |
| N06AX26 | vortioxetine | 10 mg | < 5 mg | ≥ 5 - ≤ 15 mg | > 15 mg |
| **Statins** |  |  |  |  |  |
| C10AA01 | simvastatin | 30 mg | < 15 mg | ≥ 15 - ≤ 45 mg | > 45 mg |
| C10AA03 | pravastatin | 30 mg | < 15 mg | ≥ 15 - ≤ 45 mg | > 45 mg |
| C10AA04 | fluvastatin | 60 mg | < 30 mg | ≥ 30 - ≤ 90 mg | > 90 mg |
| C10AA05 | atorvastatin | 20 mg | < 10 mg | ≥ 10 - ≤ 30 mg | > 30 mg |
| C10AA07 | rosuvastatin | 10 mg | < 5 mg | ≥ 5 - ≤ 15 mg | > 15 mg |

ᵃ ATC: Anatomical Therapeutic Chemical classification system of the world health organization (WHO); categorizes drugs by the organ/system they act on and their therapeutic, pharmacological, and chemical properties.

ᵇ DDD: Defined Daily Dose; the assumed average maintenance dose per day for a drug used for its main indication in adults (WHO).

**Online Resource Table S2. Index and marker medications (ATC) linked to their associated ADRs**

| **ADR (MedDRA notation)ᵃ** | **Index medication (ATC-code)** | **Marker medication (ATC-code)ᵇ** |
| --- | --- | --- |
| Cough (10011224) | ACE-inhibitors, plain, combinations (C09A, C09B, C09BX) | Cough and cold preparations (R05) |
| Infection (10021789) | Proton pump inhibitors (A02BC) | Intestinal anti-infectives (A07A) |
| Cough (10011224) | ACE-inhibitors, plain, combinations (C09A, C09B, C09BX) | Antibacterials for systemic use (within J01A, J01B, J01F, J01G, J01R) |
| Urinary tract infections (UTI) (10046571) | ACE-inhibitors, plain, combinations (C09A, C09B, C09BX) | Antibacterials for systemic use (within J01C, J01D, J01E, J01M, J01X) |
| Erectile dysfunction (10061461) | HMG CoA reductase inhibitors (C10AA) and in combination (C10B) | Drugs used in erectile dysfunction (G04BE) |
| Cough (10011224) | ACE-inhibitors, plain, combinations (C09A, C09B, C09BX) | Antihistamines for systemic use (R06A) |
| Sleeplessness (10041017) | HMG CoA reductase inhibitors (C10AA) and in combination (C10B) | Hypnotics and sedatives (N05C) |
| Urinary incontinence (10046543) | HMG CoA reductase inhibitors (C10AA) and in combination (C10B) | Drugs for urinary frequency and incontinence (G04BD), Drugs used in benign prostatic hypertrophy (G04C) |
| Arthritis (20000216) | ACE-inhibitors, plain, combinations (C09A, C09B, C09BX) | Anti-inflammatory and antirheumatic products, non-steroids (M01A) |
| Agitation (10001497) | HMG CoA reductase inhibitors (C10AA) and in combination (C10B) | Antipsychotics (N05A) exluding lithium (N05AN)/ Benzodiazepines (N05BA, N05CD) |
| Confusion state (10010304) | HMG CoA reductase inhibitors (C10AA) and in combination (C10B) | Antipsychotics (N05A), exluding lithium (N05AN) |
| Cough (10011224) | ACE-inhibitors, plain, combinations (C09A, C09B, C09BX) | Adrenergics, inhalants (R03A) |
| Depression (10012378) | HMG CoA reductase inhibitors (C10AA) and in combination (C10B) | Non-selective monoamine reuptake inhibitors (N06AA), Selective serotonin reuptake inhibitors (N06AB), Other antidepressants (N06AX) |
| Diuresis excessive (10013524) | Diuretics (C03) | Urologicals (G04BD) |
| Depression (10012378) | ACE-inhibitors, plain, combinations (C09A, C09B, C09BX) | Non-selective monoamine reuptake inhibitors (N06AA), Selective serotonin reuptake inhibitors (N06AB), Other antidepressants (N06AX) |
| Oedema peripheral (10030124) | Antiinflammatory and antirheumatic products, non-steroids (M01A) | High-ceiling diuretics (C03C) |
| Depression (10012378) | Dihydropyridines (C08C, C08E, C08G, C09DB), excluding C09BB10 | Non-selective monoamine reuptake inhibitors (N06AA), Selective serotonin reuptake inhibitors (N06AB), Other antidepressants (N06AX) |
| Urinary incontinence (10046543) | Antidepressants (N06A) | Drugs for urinary frequency and incontinence (G04BD, Drugs used in benign prostatic hypertrophy (G04C) |

ᵃ MedDRA: Medical Dictionary for Regulatory Activities; an international standard terminology maintained by ICH for coding adverse events. Preferred Terms (PT) and their numeric codes are shown in parentheses.

ᵇ ATC: Anatomical Therapeutic Chemical classification system of the WHO; categorizes drugs by the organ/system they act on and their therapeutic, pharmacological, and chemical properties.

**Online Resource Table S3. Overview of excluded cascades and the corresponding reasons for exclusion.**

| **Cascade (Index–ADR–Marker)** | **Reason for Exclusion** |
| --- | --- |
| Amiodarone - hypothyroidism- thyroid hormones | Insufficient user data |
| Lithium- tremor- propranolol | Indication of a dose-response relationship |
| HMG CoA reductase inhibitors and in combination- cognitive impairment- anti-dementia drugs | Indication of a dose-response relationship |
| Lithium - parkinsonism - tertiary amines, dopaminergic agents | Insufficient user data |
| Lithium- hypothyroidism- thyroid hormones | Insufficient user data |
| Antipsychotics (excluding lithium)- hyperprolactinaemia & oligomenorrhea (primary)- prolactin inhibitors | Indication of a dose-response relationship |
| Antipsychotics (excluding lithium)- parkinsonism- tertiary amines, dopaminergic agents | Indication of a dose-response relationship |
| ACE-inhibitors, plain, combinations- erectile dysfunction- drugs used in erectile dysfunction | Insufficient user data |
| Angiotensin II receptor blockers, plain and combinations- erectile dysfunction- drugs used in erectile dysfunction | Insufficient user data |
| Dihydropyridines- erectile dysfunction- drugs used in erectile dysfunction | Insufficient user data |
| Beta blocking agents- erectile dysfunction- drugs used in erectile dysfunction | Insufficient user data |
| Low-ceiling diuretics- gout- anti-gout medication | Insufficient user data |
| Selective calcium channel blockers with direct cardiac effects- erectile dysfunction- drugs used in erectile dysfunction | Insufficient user data |
| Low-ceiling diuretics- gout- anti-gout medication | Indication of a dose-response relationship |
| High-ceiling diuretics- erectile dysfunction- drugs used in erectile dysfunction | Insufficient user data |
| Antiepileptics, other- urinary tract infections- antibacterials for systemic use | Cascade with tapering schedule of the index medication |
| Antidepressants- migraine- other analgesics and antipyretics | Indication of a dose-response relationship |
| Selective calcium channel blockers with direct cardiac effects– oedema peripheral- high ceiling diuretics | Insufficient user data |
| Antidepressants- parkinsonism- tertiary amines, dopaminergic agents | Indication of a dose-response relationship |
| Beta-blocking agents- depression- non-selective monoamine reuptake inhibitors, selective serotonin reuptake inhibitors | Indication of a dose-response relationship |
| Antiepileptics- oedema peripheral- high ceiling diuretics | Indication of a dose-response relationship |
| HMG CoA reductase inhibitors and in combination- arrhythmia- antithrombotic agents | Indication of a dose-response relationship |

**Elaboration on the reasons for exclusion:**

**Insufficient user data:** The cascade did not include a sufficient number of users. Sufficient total users are defined as more than 3,000 users.

**Indication of a dose-response relationship:** Literature suggests that a dose-response relationship has been is plausible or observed. Therefore, we did not conduct further analysis on this, as our focus was on cascades where the dose-response relationship was either unknown or not observed.

**Cascade with tapering schedule:** Prescribing cascades with a titration or tapering regimen were excluded, as no daily dose could be calculated.

**Online Resource Table S4. Overview per potential prescribing cascade of proportion of excluded dispensings and patients due to missing information on the dose.**

| Potential prescribing cascade | % excluded dispensings | % excluded patients |
| --- | --- | --- |
| Dihydropyridine calcium channel blockers potentially causing oedema followed by high-ceiling diuretics(positive control) | 1.7 | 0.8 |
| ACEI potentially causing cough followed by inhaled adrenergics | 1.9 | 1.0 |
| ACEI potentially causing cough followed by antibacterials for systemic use | 0.2 | 5.4 |
| ACEI potentially causing cough followed by cough and cold preparations | 1.6 | 0.7 |
| ACEI potentially causing cough followed by antihistamines for systemic use | 1.6 | 0.8 |
| ACEI potentially causing urinary tract infections followed by antibacterials for systemic use | 1.3 | 0.9 |
| ACEI potentially causing arthritis followed by systemic NSAIDs/anti-rheumatics | 0.2 | 6.1 |
| ACEI potentially causing depression followed by antidepressants | 0.5 | 5.7 |
| Statins potentially causing erectile dysfunction followed by drugs for erectile dysfunction | 0.3 | 0.2 |
| Statins potentially causing urinary incontinence followed by drugs for incontinence | 1.6 | 0.9 |
| Statins followed by antidepressants | 1.3 | 0.8 |
| Statins potentially causing agitation followed by antipsychotics | 1.6 | 0.9 |
| Statins potentially causing confusion followed by antipsychotics | 0.4 | 0.7 |
| Statins potentially causing sleeplessness followed by hypnotics/sedatives | 1.5 | 1.1 |
| Antidepressants potentially causing urinary incontinence followed by drugs for incontinence | 2.5 | 5.0 |
| Dihydropyridine CCB potentially causing depression followed by antidepressants | 0.3 | 2.2 |
| Thiazide & loop diuretics potentially causing diuresis followed by urologicals | 1.1 | 1.4 |
| Systemic NSAIDs potentially causing peripheral oedema followed by diuretics | 26.7 | 27.7 |
| Proton pump inhibitors potentially causing infection followed by intestinal anti-infectives | 4.2 | 4.5 |

Abbreviations: ACEI: Angiotensin Converting Enzyme inhibitor; CCB: calcium channel blocker; NSAID: non-steroidal anti-inflammatory drug.

**Online resource Table S5: sensitivity analyses with different cut-offs used to define low, medium and high dose.**

| Cascade | Total | IM | MI | **Null-effect** | **cSR** | **aSR [95% CI]** |
| --- | --- | --- | --- | --- | --- | --- |
| **Dihydropyridine calcium channel blockers potentially causing oedema followed by high-ceiling diuretics(positive control)** | | | | | | |
| Overall | 10113 | 6667 | 3446 | 1.07 | 1.93 | 1.82 [1.77 – 1.86] |
| Low dose | 306 | 189 | 117 | 1.06 | 1.62 | 1.53 [1.30 – 1.76]c |
| Medium dose | 6812 | 4352 | 2460 | 1.05 | 1.77 | 1.68 [1.63 – 1.73]b |
| High dose | 2995 | 2126 | 869 | 1.09 | 2.45 | 2.24 [2.16 – 2.31]b, c |
| **ACEI potentially causing cough followed by inhaled adrenergics** | | | | | | |
| Overall | 14927 | 8308 | 6619 | 1.03 | 1.26 | 1.22 [1.19 – 1.25] |
| Low dose | 4860 | 2512 | 2348 | 1.01 | 1.07 | 1.06 [1.00 – 1.11]d |
| Medium dose | 7258 | 4062 | 3196 | 1.03 | 1.27 | 1.23 [1.19 – 1.28]d |
| High dose | 2809 | 1734 | 1075 | 1.07 | 1.61 | 1.51 [1.44 – 1.59]d |
| **ACEI potentially causing cough followed by antibacterials for systemic use** | | | | | | |
| Overall | 11646 | 8043 | 3603 | 1.11 | 2.23 | 2.00 [1.96 – 2.04] |
| Low dose | 3737 | 2486 | 1251 | 1.09 | 1.99 | 1.82 [1.75 – 1.89]d |
| Medium dose | 5720 | 3944 | 1776 | 1.12 | 2.22 | 1.98 [1.92 – 2.03]d |
| High dose | 2189 | 1613 | 576 | 1.14 | 2.80 | 2.47 [2.37 – 2.56]d |
| **ACEI potentially causing cough followed by cough and cold preparations** | | | | | | |
| Overall | 19173 | 14215 | 4958 | 1.12 | 2.87 | 2.55 [2.52 – 2.58] |
| Low dose | 6294 | 4560 | 1734 | 1.10 | 2.63 | 2.39 [2.33 – 2.44]d |
| Medium dose | 9575 | 7147 | 2428 | 1.13 | 2.94 | 2.60 [2.55 – 2.65]d |
| High dose | 3304 | 2508 | 796 | 1.15 | 3.15 | 2.75 [2.67 – 2.83]d |
| **ACEI potentially causing cough followed by antihistamines for systemic use** | | | | | | |
| Overall | 11475 | 7294 | 4181 | 1.06 | 1.74 | 1.64 [1.61 – 1.68] |
| Low dose | 3606 | 2197 | 1409 | 1.05 | 1.56 | 1.49 [1.42 – 1.56]d |
| Medium dose | 5907 | 3737 | 2170 | 1.06 | 1.72 | 1.62 [1.57 – 1.68]d |
| High dose | 1962 | 1360 | 602 | 1.09 | 2.26 | 2.07 [1.98 – 2.17]d |
| **ACEI potentially causing urinary tract infections followed by antibacterials for systemic use** | | | | | | |
| Overall | 32673 | 21997 | 10676 | 1.09 | 2.06 | 1.89 [1.87 – 1.91] |
| Low dose | 10892 | 7092 | 3800 | 1.07 | 1.87 | 1.74 [1.70 – 1.78]d |
| Medium dose | 15624 | 10436 | 5188 | 1.09 | 2.01 | 1.84 [1.81 – 1.88]d |
| High dose | 6157 | 4469 | 1688 | 1.12 | 2.65 | 2.36 [2.31 – 2.42]d |
| **ACEI potentially causing arthritis followed by non-steroid anti-inflammatory / anti-rheumatic medications*** | | | | | | |
| Overall | 23151 | 13594 | 9557 | 1.06 | 1.42 | 1.34 [1.32 – 1.37] |
| Low dose | 6442 | 3690 | 2752 | 1.05 | 1.34 | 1.28 [1.23 – 1.33]c |
| Medium dose | 12299 | 7095 | 5204 | 1.06 | 1.36 | 1.29 [1.26 – 1.33]b |
| High dose | 4410 | 2809 | 1601 | 1.09 | 1.75 | 1.61 [1.55 – 1.67]b, c |
| **ACEI potentially causing depression followed by antidepressants**** | | | | | | |
| Overall | 9719 | 5210 | 4509 | 1.02 | 1.16 | 1.13 [1.09 – 1.17] |
| Low dose | 3083 | 1595 | 1488 | 1.01 | 1.07 | 1.07 [1.00 – 1.14]c |
| Medium dose | 4743 | 2527 | 2216 | 1.02 | 1.14 | 1.12 [1.06 – 1.17]b |
| High dose | 1893 | 1088 | 805 | 1.05 | 1.35 | 1.29 [1.20 - 1.38]b, c |
| **Statins potentially causing erectile dysfunction followed by drugs used in erectile dysfunction** | | | | | | |
| Overall | 4933 | 3267 | 1666 | 1.08 | 1.96 | 1.81 [1.75 – 1.87] |
| Low dose | 546 | 371 | 175 | 1.08 | 2.12 | 1.96 [1.78 – 2.14] |
| Medium dose | 3470 | 2264 | 1206 | 1.08 | 1.88 | 1.74 [1.67 – 1.81]b |
| High dose | 917 | 632 | 285 | 1.10 | 2.22 | 2.02 [1.88 – 2.16]b |
| **Statins potentially causing urinary incontinence followed by drugs for urinary frequency and incontinence** | | | | | | |
| Overall | 11808 | 7072 | 4736 | 1.05 | 1.49 | 1.42 [1.38 – 1.46] |
| Low dose | 1545 | 908 | 637 | 1.04 | 1.43 | 1.37 [1.27 – 1.47] |
| Medium dose | 8279 | 4962 | 3317 | 1.05 | 1.50 | 1.42 [1.38 – 1.46] |
| High dose | 1984 | 1202 | 782 | 1.06 | 1.54 | 1.46 [1.37 – 1.55] |
| **Statins potentially causing depression followed by antidepressants**** | | | | | | |
| Overall | 16788 | 9227 | 7561 | 1.03 | 1.22 | 1.18 [1.15 – 1.21] |
| Low dose | 2135 | 1126 | 1009 | 1.02 | 1.12 | 1.10 [1.01 – 1.18] |
| Medium dose | 11979 | 6592 | 5387 | 1.03 | 1.22 | 1.19 [1.15 – 1.22] |
| High dose | 2674 | 1509 | 1165 | 1.04 | 1.30 | 1.25 [1.17 – 1.32] |
| **Statins potentially causing agitation followed by antipsychotics / benzodiazepines** | | | | | | |
| Overall | 25748 | 15124 | 10624 | 1.05 | 1.42 | 1.35 [1.33 – 1.38] |
| Low dose | 3461 | 2043 | 1418 | 1.05 | 1.44 | 1.37 [1.30 – 1.44] |
| Medium dose | 18107 | 10620 | 7487 | 1.05 | 1.42 | 1.35 [1.32 – 1.38] |
| High dose | 4180 | 2461 | 1719 | 1.05 | 1.43 | 1.36 [1.30 – 1.43] |
| **Statins potentially causing confusion followed by anti-psychotics** | | | | | | |
| Overall | 4524 | 2586 | 1938 | 1.04 | 1.33 | 1.28 (1.22 – 1.34) |
| Low dose | 509 | 309 | 200 | 1.04 | 1.55 | 1.48 (1.31 – 1.66)e |
| Medium dose | 3357 | 1883 | 1474 | 1.04 | 1.28 | 1.23 (1.16 – 1.30)e |
| High dose | 658 | 394 | 264 | 1.05 | 1.49 | 1.42 (1.26 – 1.58) |
| **Statins potentially causing sleeplessness followed by hypnotics and sedatives** | | | | | | |
| Overall | 15033 | 9088 | 5945 | 1.06 | 1.53 | 1.45 [1.41 – 1.48] |
| Low dose | 1998 | 1175 | 823 | 1.05 | 1.43 | 1.36 [1.27 – 1.45] |
| Medium dose | 10649 | 6465 | 4184 | 1.06 | 1.55 | 1.46 [1.42 – 1.50] |
| High dose | 2386 | 1448 | 938 | 1.05 | 1.54 | 1.47 [1.38 – 1.55] |
| **Antidepressants potentially causing urinary incontinence followed by drug for urinary frequence incontinence** | | | | | | |
| Overall | 6400 | 3349 | 3051 | 1.01 | 1.10 | 1.09 [1.04 – 1.13] |
| Low dose | 3502 | 1586 | 1916 | 0.98 | 0.83 | 0.85 [0.78 – 0.91]d |
| Medium dose | 2296 | 1346 | 950 | 1.04 | 1.42 | 1.36 [1.28 – 1.44]d |
| High dose | 602 | 417 | 185 | 1.10 | 2.25 | 2.05 [1.87 – 2.22]d |
| **Dihydropyridine calcium channel blockers potentially causing depression followed by antidepressants**** | | | | | | |
| Overall | 8849 | 4702 | 4147 | 1.02 | 1.13 | 1.12 [1.07 – 1.16] |
| Low dose | 317 | 145 | 172 | 0.99 | 0.84 | 0.85 [0.63 – 1.07]c |
| Medium dose | 6388 | 3331 | 3057 | 1.01 | 1.09 | 1.08 [1.03 – 1.13]b |
| High dose | 2144 | 1226 | 918 | 1.04 | 1.34 | 1.29 [1.20 – 1.37]b, c |
| **Thiazide and loop diuretics potentially causing diuresis followed by urologicals** | | | | | | |
| Overall | 3201 | 1741 | 1460 | 1.04 | 1.19 | 1.15 [1.08 – 1.22] |
| Low dose | 1710 | 938 | 772 | 1.04 | 1.22 | 1.17 [1.08 – 1.27] |
| Medium dose | 651 | 359 | 292 | 1.07 | 1.23 | 1.15 [1.00 – 1.31] |
| High dose | 840 | 444 | 396 | 1.02 | 1.12 | 1.10 [0.96 – 1.23] |
| **Systemic non-steroid antiinflammatory and antirheumatic medications* potentially causing peripheral oedema followed by high-ceiling diuretics** | | | | | | |
| Overall | 8170 | 4407 | 3763 | 1.01 | 1.17 | 1.16 [1.12 – 1.21] |
| Low dose | 651 | 353 | 298 | 1.02 | 1.18 | 1.17 [1.01 – 1.32] |
| Medium dose | 2933 | 1531 | 1402 | 1.00 | 1.09 | 1.10 [1.02 – 1.17] |
| High dose | 4586 | 2523 | 2063 | 1.01 | 1.22 | 1.21 [1.15 – 1.27] |
| **Proton pump inhibitors potentially causing infection followed by intestinal antiinfectives** | | | | | | |
| Overall | 7909 | 5810 | 2099 | 1.10 | 2.77 | 2.51 [2.46 – 2.56] |
| Low dose | 651 | 460 | 191 | 1.09 | 2.41 | 2.22 [2.05 – 2.38]e |
| Medium dose | 4638 | 3432 | 1206 | 1.10 | 2.85 | 2.58 [2.52 – 2.65]e |
| High dose | 2620 | 1918 | 702 | 1.11 | 2.73 | 2.47 [2.38 – 2.55] |

Abbreviations: cSR= crude sequence ratio. aSR= adjusted sequence ratio, SD: standard deviation, ACEI: angiotensin-converting enzyme inhibitor, NSAID: non-steroidal anti-inflammatory drug.
a; no overlap between low and medium dose.
b; no overlap between medium and high dose.
c; no overlap between low and high dose.
d; no overlap between all three dose-categories.

e: no overlap between low and medium dose, but high dose category does not reflect an aSR increase across dose categories.

* Non-steroid antiinflammatory and antirheumatic medications M01A.

** Antidepressants (N06AA, N06AB, N06AX).
